# Association of Multidimensional Poverty With Dementia in Adults Aged 50 Years or Older in South Africa

**DOI:** 10.1001/jamanetworkopen.2022.4160

**Published:** 2022-03-25

**Authors:** Jean-Francois Trani, Jacqueline Moodley, May Thu Thu Maw, Ganesh M. Babulal

**Affiliations:** 1 Brown School, Washington University, St Louis, Missouri; 2 Department of Psychology, Faculty of Humanities, University of Johannesburg, Johannesburg, South Africa; 3 Center for Social Development in Africa, University of Johannesburg, Johannesburg, South Africa; 4 Department of General Internal Medicine, Johns Hopkins University School of Medicine, Baltimore, Maryland; 5 Department of Neurology, Washington University School of Medicine, St Louis, Missouri; 6 Department of Clinical Research and Leadership, The George Washington University School of Medicine and Health Sciences, Washington, DC

## Abstract

**Importance:**

Limited research exists investigating the association between multidimensional poverty and dementia in low-and middle-income countries (LMICs).

**Objective:**

To investigate the association between multidimensional poverty and dementia among adults aged 50 years or older living in South Africa.

**Design, Setting, and Participants:**

This cross-sectional study was conducted in Soweto, Johannesburg, South Africa, between November 11, 2019, and February 28, 2020. Participants included 227 adults aged 50 years or older. Data analysis was concluded from August 1 to 30, 2021.

**Exposures:**

Multidimensional poverty included 7 dimensions that are central to well-being (education, health, economic activity, living standards, social participation, fair treatment, and psychological well-being) and 11 indicators of deprivation within those dimensions (limited access to education; severe limitation of activity; difficulty functioning; unemployment; deprivation of access to running water, electricity, and a flush toilet; lack of involvement in community groups; discrimination; depression; and decreased self-esteem).

**Main Outcomes and Measures:**

The 8-item Interview to Differentiate Aging and Dementia (Assessing Dementia 8 [AD8]) and the Rowland Universal Dementia Assessment Scale (RUDAS) were used to assess dementia. Level and depth of poverty were compared between adults with no dementia and those with a score above the threshold for either the AD8 or the RUDAS, or for both the AD8 and the RUDAS, adjusting for gender, age group, and marital status. Correlation analyses assessed the overlap of dimensions of deprivation. Associations between dementia and multidimensional poverty were investigated using a multivariable logistic regression model.

**Results:**

A total of 227 adults (146 women [64.3%]; mean [SD] age, 63.7 [0.5] years) were included in the study; 101 (44.5%) had dementia identified by the AD8, 14 (6.2%) had dementia identified by the RUDAS, and 50 (22.0%) had dementia identified by both the AD8 and the RUDAS. More men than women did not have dementia (26 of 81 [32.1%] vs 36 of 146 [24.7%]), and 33 of 165 adults with dementia (20.0%) compared with 6 of 62 adults (9.7%) without dementia were found to be deprived in 4 dimensions or more. The difference between adults with and adults without dementia in the Multidimensional Poverty Index for deprivation in 4 dimensions was 145.8% for dementia identified by both the AD8 and the RUDAS and 118.2% for dementia identified by either the AD8 or the RUDAS. Education, health, and employment were the main contributors to the adjusted poverty head count ratio. Multidimensional poverty was strongly associated with dementia as measured by the AD8 and the RUDAS (adjusted odds ratio [OR], 2.31; 95% CI, 1.08-4.95), with higher odds for older women (OR, 2.03; 95% CI, 1.00-4.12) or those living in large households (for each additional household member: OR, 1.27; 95% CI, 1.05-1.53).

**Conclusions and Relevance:**

This study suggests that the prevalence and depth of poverty were higher among adults with dementia. A lack of education, poor health, and unemployment were major dimensions of poverty that were associated with a higher prevalence of dementia. Long-term interventions beginning early in life may affect social determinants of health through targeted structural policies (eg, access to quality education and health care) and prevent dementia later in life.

## Key Points

**Question:**

Is multidimensional poverty associated with dementia in older adults in low- and middle-income countries?

**Findings:**

In this cross-sectional study of 227 adults aged 50 years or older living in Soweto, Johannesburg, South Africa, 20% of adults with dementia were poor compared with 10% of those without dementia, with a difference of 118% on the Multidimensional Poverty Index. The risk of dementia was 2.31 times higher for adults who were multidimensionally poor.

**Meaning:**

These results suggest that public policies addressing factors associated with poverty may be helpful to delay the onset and reduce the prevalence of dementia among older adults.

## Teaser

This cross-sectional study investigates the association between multidimensional poverty and dementia among adults aged 50 years or older living in South Africa.

## Introduction

Dementia is a global health challenge that affects high-income countries but has profound repercussions for low- and middle-income countries (LMICs).^1^ The World Health Organization estimates that adults with all-cause dementia (estimates: 5%-7% for those ≥60 years)^2^ will more than triple to reach 152 million by 2050, with at least 60% of those individuals living in LMICs.^3^ This increase is associated with the overall growth of the population and the aging demographic.^4^

Dementia-related costs will increase during the next 3 decades, and LMICs will be responsible for shouldering this economic burden.^5^ Families will have to absorb these costs because formal geriatric care is in its infancy in LMICs.^6^ Older adults are both the recipients and the providers of informal care, in which the reciprocity of care between generations has been shown to improve well-being.^7,8^ This advantage disappears with dementia. Older adults with dementia become unable to provide care for other family members, a major problem in societies in which few formal systems of care exist. Similarly, adults with dementia need more financial, social and emotional, and physical support from other family members. This increased need directly affects the caregiver’s ability to work and attend school, especially because many children are tasked with providing care.^9^

The 2020 *Lancet* Commission on dementia prevention, intervention, and care identified 12 potentially modifiable risk factors for dementia, including poor education, hypertension, depression, low social contact, smoking, air pollution, and traumatic brain injury.^10^ It is well documented that poor social determinants of health are directly associated with disease.^11,12,13,14,15,16^ However, to our knowledge, limited research exists investigating how social determinants of health are associated with dementia. Context-specific studies using multidimensional poverty measures are required to better understand this association.^13,17,18,19^ A multidimensional approach to poverty encompassing various components of well-being is measured in terms of individuals’ functioning and capabilities instead of resources or utility. This approach offers a more precise account of risk factors that eventually can trigger multiple conditions, including dementia, at a later stage in life and offers insight into how to improve care and policy.^20,21^ For instance, multidimensional poverty has been shown to be associated with chronic health conditions.^22^ Furthermore, various dimensions of deprivation, including chronic infectious diseases, poor health status, malnutrition, prenatal stress,^23^ poor mental health, low access to health care, low level of literacy,^24,25^ and lack of occupation,^26,27^ are associated with income-based poverty and have been shown to be associated with aging and dementia. In LMICs, identifying specific dimensions of deprivation may help target interventions and public policies to mitigate an immediate risk of cognitive impairment,^28^ support older adults and their caregivers,^29^ and slow cognitive decline.

We searched PubMed and Scopus for scientific publications about poverty and dementia in LMICs since 2000. We used the following combination of search terms: *dementia*, *Alzheimer*, Alzheimer disease*, *cognitive disorders* AND *poor* OR *poverty*, and *social determinants of health*. The literature on dementia in LMICs is scarce, and little is known about the quantitative association between dementia and multidimensional poverty in LMICs. One study investigated risk prediction models of dementia applied to LMICs.^30^ One study showed that individuals with Alzheimer disease have a lower level and smaller range of capabilities.^31^

Since the end of apartheid nearly 3 decades ago, overall poverty levels have decreased in South Africa, the second-largest economy in sub-Saharan Africa. However, more than half the population continues to live with an income under the poverty line, and since 1994, almost 2.5 million South Africans, many of whom are Black or of mixed race, became poor.^32^ Moreover, older age is widely recognized as the main nonmodifiable risk factor for dementia in South Africa and LMICs.^33^ Estimates from studies conducted in South Africa indicate that the prevalence of dementia ranges from 3.8% to 11.0% for adults aged 65 years or older.^33,34,35^ In this study, we examined multidimensional poverty and dementia in older adults in Soweto, South Africa. Our objective was to examine the association between multidimensional poverty and dementia and the role of various dimensions of deprivation in overall poverty. Using multidimensional poverty measures, we hypothesized that social determinants of health are positively associated with dementia in older adults.

## Methods

Ethical approval for this cross-sectional study was obtained from the Faculty of Humanities Research Ethics Committee at the University of Johannesburg in South Africa and the Human Research Protection Office of Washington University in St Louis, Missouri. Written informed consent was obtained from all participants except for those who lacked the capacity to provide it; for these participants, verbal assent was obtained using a simplified text, and their caregiver also provided written consent. This study adhered to the Strengthening the Reporting of Observational Studies in Epidemiology (STROBE) reporting guideline for standard reporting in cross-sectional studies.

### Study Design and Sample

**Figure 1.**
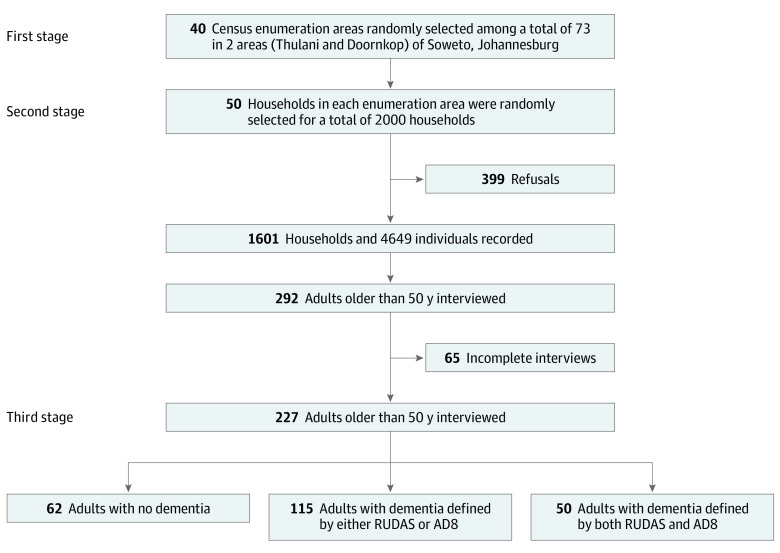
Participant Recruitment AD8 indicates 8-item Interview to Differentiate Aging and Dementia; and RUDAS, Rowland Universal Dementia Assessment Scale.

Participants were first selected from a 2-stage, random cluster cross-sectional study conducted in June 2017 in 40 of 73 census enumeration areas in 2 wards of Soweto, Thulani, and Doornkop in Johannesburg, South Africa. A second round of interviews was conducted between November 11, 2019, and February 28, 2020, among 292 adults aged 50 years or older. We excluded 65 individuals (22.3%) owing to incomplete interviews. The final sample included 227 older adults (Figure 1). Instruments went through forward and backward translation into Zulu (the dominant language in Soweto), with different translators to ensure accuracy, and were tested and validated.

### Dementia Outcome

The 8-item Interview to Differentiate Aging and Dementia (Assessing Dementia 8 [AD8])^36^ and the Rowland Universal Dementia Assessment Scale (RUDAS)^37^ were used to assess dementia. In the absence of access to imaging or cerebrospinal fluid biomarkers, a common problem in many LMICs (including sub-Saharan Africa),^38^ screening tools are the best way to identify those at risk of dementia. Data on demographic characteristics, health conditions, educational level, employment, livelihood, depression, self-esteem, stigma, social cohesion, and social participation were obtained. A field supervisor and 5 enumerators were trained over 2 days on survey concepts and goals, questionnaires, and interviewing techniques.

### Multidimensional Poverty Measures

To measure multidimensional poverty, we included 7 dimensions that are central to well-being: education, health, economic activity, living standards, social participation, fair treatment, and psychological well-being (eTable 1 in the Supplement). Each dimension contained indicators identified in the literature as crucial to human development.

Education is associated with one’s ability to gain employment and earn an income, which may have affected participants in this study who were exposed to the Bantu education system, notoriously known to limit Black African individuals from accessing socioeconomic opportunities.^39^ Study participants were considered deprived if they had access only to primary education, a threshold shared with the South African Multidimensional Poverty Index.^40^

Any severe activity limitation or functioning difficulty in 6 areas was considered as the cutoff for deprivation of health.^41^ Disability considerably limits an individual’s access to practical opportunities^42,43^ and is associated with a higher incidence of disease and poverty in South Africa.^44,45^

Unemployment is an indicator of deprivation^46^ and economic hardship^46^ and a major challenge in South Africa, with one-third of the population unemployed.^47^ The cutoff was used if an adult was unemployed, looking for a job, or not looking for a job because the participant was discouraged or could not afford the cost of seeking work or the wages offered were too low.

Household living standards were composed of 3 indicators (water pipe, electricity, and flush toilet), for which deprivation within the compound was the cutoff, which are important indicators of quality of life in South Africa.^48,49^

Involvement in community groups has been shown to enhance life satisfaction.^50^ Social participation, resulting in community interconnectedness, is a central feature of *ubuntu* (a philosophy that emphasizes the importance of community) in South Africa, and study participants who were not involved in any group were considered deprived.^51,52^ Discrimination and stigma were measured using the validated 22-item Unfair Treatment subscale of the Discrimination and Stigma Scale.^53^ Content and face validity tests were conducted. Moderate discrimination was the cutoff.^53,54^

Finally, measures of depression and self-esteem represented deprivation of psychological well-being and are often associated with poverty, particularly among older adults.^55,56,57,58^ Depression was measured using the 10-item Center for Epidemiologic Studies Depression Scale Revised (CESD-R-10),^59^ already validated in South Africa.^60^ A score of 10 or higher was the established cutoff. Self-esteem was measured using the 10-item Rosenberg Self-Esteem Scale, validated in South Africa,^61,62^ with a score below 15 as the established cutoff. Studies have shown that aging is associated with a decrease in self-esteem^63^ and with multidimensional poverty.^64^ There is no known association between dementia and low levels of self-esteem. However, dementia may lead to a sense of insecurity, affecting a person’s ability to make decisions and to maintain personal relationships, employment, health, and finances.^58^ As a result, inclusion of self-esteem in the multidimensional poverty measure is important.

### Statistical Analysis

Statistical analysis was conducted from August 1 to 30, 2021. We used an unmatched, multidimensional poverty measure to identify differences in levels of poverty comparing those with and those without dementia. Dimensions of deprivation were independently assessed, and the method focused on dimensional shortfalls.^65^ We aggregated dimensions of multidimensional poverty measures that consisted of 2 cutoffs: 1 for each dimension and 1 associated with cross-cutting dimensions. Older adults were considered deprived if they fell below the cutoff on a given dimension. The second poverty cutoff determined the number of dimensions in which an older adult had to be deprived to be deemed multidimensionally poor.

One-way analyses examined differences in the poverty level between adults with no dementia and those with a score above the threshold for either the AD8 or the RUDAS, or for both AD8 and RUDAS, and comparing gender, age group, and marital status. We adjusted for post hoc pairwise comparisons.^66^ We also performed a correlation analysis to assess the overlap of dimensions of deprivation.

We measured 3 indicators: (1) the poverty head count that indicates the number of older adults who lived below the poverty line; (2) the mean deprivation share, which is the mean number of dimensions of deprivation experienced by each older adult who lived below the poverty line; and (3) the adjusted head count ratio, which is the product of the poverty head count and the mean deprivation share. The adjusted head count ratio denotes the intensity of poverty.^65^

Robustness was examined via 2 different weighting structures: (1) equal weight of 1 for every indicator (equal indicators weight) in each dimension and (2) equal weight associated with the 7 dimensions and equal weight for each indicator within each dimension (equal nested weight). In a dimension with 1 indicator (eg, education), the weight of the indicator is 1. However, the weight of each indicator is 1 of 3 in a dimension with 3 indicators (eg, living standards). Both methods provided consistent results.

We calculated unadjusted and adjusted logistic regression models to identify the association between dementia and multidimensional poverty. The binary outcome compared adults with and adults without dementia as determined by either the AD8 or the RUDAS. Multidimensional poverty exposure was defined as being deprived in 4 dimensions, in which each adult is deprived corresponding to the highest gap between adults with and adults without dementia and a prevalence of poverty of 17% among all adults. This was based on estimates of 25.4% of urban South African individuals living below the national lower bound of the poverty line.^67^ We investigated whether multidimensional poverty was associated with dementia in older adults. Models adjusted for gender (man or woman), age (continuous), marital status (living alone or living with a partner), and household size (continuous). All *P* values were from 2-tailed tests, and results were deemed statistically significant at *P* < .05. Missing values (n = 10) were treated as being missing completely at random. Data were analyzed using Stata, version 16.0 (StataCorp).

## Results

### Participant Characteristics

Among 227 respondents (146 women [64.3%]; mean [SD] age, 63.7 [0.5] years), there were 62 adults (27.3%) without dementia, 101 adults (44.5%) with dementia identified by the AD8, 14 (6.2%) with dementia identified by the RUDAS, and 50 (22.0%) with dementia identified by both the AD8 and the RUDAS (Table 1). Fewer women (24.7% [36 of 146]) had no dementia compared with men (32.1% [26 of 81]). The proportion of adults without dementia was similar from 50 to 79 years of age (50-59 years, 21 of 80 [26.3%]; 60-69 years, 28 of 97 [28.9%]; 70-79 years, 11 of 40 [27.5%]), but this proportion decreased with older age (≥80 years, 2 of 10 [20.0%]). Dementia identified using the RUDAS was more prevalent with age: 27.5% (22 of 80) of adults aged 50 to 59 years, 23.7% (23 of 97) of those aged 60 to 69 years, 37.5% (15 of 40) of those aged 70 to 79 years, and 40.0% (4 of 10) of those aged 80 years or older.

**Table 1.** Sample Characteristics

Characteristic	Adults, No. (%)			*P* value
	No CID (n = 62)^a^	CID defined by either RUDAS or AD8 (n = 115)^b^	CID defined by both RUDAS and AD8 (n = 50)^c^	
Gender				
Male	26 (41.9)	40 (34.8)	15 (30.0)	.41
Female	36 (58.1)	75 (65.2)	35 (70.0)	
Age, y				
50-59	21 (33.9)	42 (36.5)	17 (34.0)	.82
60-69	28 (45.2)	50 (43.5)	19 (38.0)	
≥70	13 (21.0)	23 (20.0)	14 (28.0)	
Marital status, No./total No. (%)				
Living with partner	23/58 (39.7)	58/110 (52.7)	19/47 (40.4)	.17
Living alone	35/58 (60.3)	52/110 (47.3)	28/47 (59.6)	

Abbreviations: AD8, 8-item Interview to Differentiate Aging and Dementia; CID, cognitive impairment disorder; RUDAS, Rowland Universal Dementia Assessment Scale.

^a^ No cognitive impairment defined by both AD8 and RUDAS.

^b^ Cognitive impairment defined by either AD8 scores or RUDAS scores. AD8: cognitive impairment present if a score of 2 or more is observed in the AD8 questions; RUDAS: a score of 22 or less should be considered as possible cognitive impairment.

^c^ Cognitive impairment defined by both AD8 scores and RUDAS scores. AD8: cognitive impairment present if a score of 2 or more is observed in the AD8 questions; RUDAS: a score of 22 or less should be considered as possible cognitive impairment.

**Figure 2.**
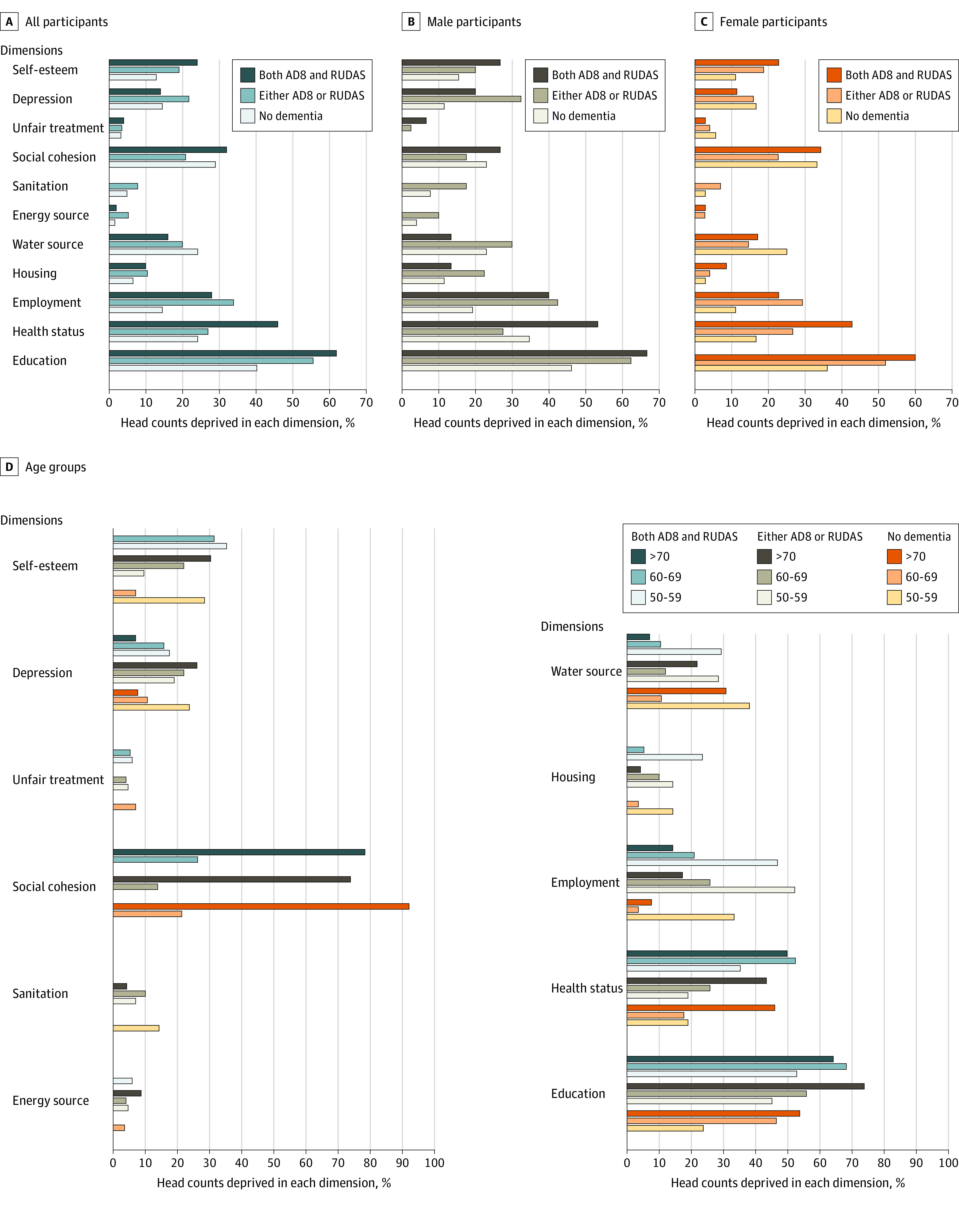
Poverty Head Counts by Dementia Status, Gender, and Age Groups AD8 indicates 8-item Interview to Differentiate Aging and Dementia; and RUDAS, Rowland Universal Dementia Assessment Scale.

There was a higher prevalence of deprivation among adults with dementia (particularly when considering those identified by both the AD8 and the RUDAS) than among adults without dementia. This finding was particularly profound for the dimensions of education (those with dementia, 31 of 50 [62.0%]; those without dementia, 25 of 62 [40.3%]; difference, 21.7 percentage points), health status (those with dementia, 23 of 50 [46.0%]; those without dementia, 15 of 62 [24.2%]; difference, 21.8 percentage points), and employment (those with dementia, 14 of 60 [23.3%]; those without dementia, 9 of 62 [14.5%]; difference, 8.85 percentage points). Differences were nonsignificant for lack of self-esteem (those with dementia, 12 of 50 [24.0%]; those without dementia, 8 of 62 [12.9%]; difference, 11.1 percentage points) and reversed for clean water (those with dementia, 8 of 50 [16.0%]; those without dementia, 15 of 62 [24.2%]; difference, −8.2 percentage points), sanitation (those with dementia, 0 of 50; those without dementia, 3 of 62 [4.8%]; difference, −4.8 percentage points), and depression (those with dementia, 7 of 50 [14.0%]; those without dementia, 9 of 62 [14.5%]; difference, −0.5 percentage points; only for dementia identified by both the RUDAS and the AD8) (Figure 2; eTable 2A-2C in the Supplement). Deprivation was higher for men and women with dementia for the dimensions of education (men: with dementia, 10 of 15 [66.7%]; without dementia, 12 of 26 [46.2%]; difference, 20.5 percentage points; women: with dementia, 21 of 35 [60.0%]; without dementia, 13 of 36 [36.1%]; difference, 23.9 percentage points), health (men: with dementia, 8 of 15 [53.3%]; without dementia, 9 of 26 [34.6%]; difference, 18.7 percentage points; women: with dementia, 15 of 35 [42.9%]; without dementia, 6 of 36 [16.7%]; difference, 26.2 percentage points) and employment (men: with dementia, 6 of 15 [40.0%]; without dementia, 5 of 26 [19.2%]; difference, 20.8 percentage points; and women: with dementia, 8 of 35 [22.9%]; without dementia, 4 of 36 [11.1%]; difference, 11.8 percentage points) compared with those without dementia, but was lower for the dimensions of access to clean water (men: with dementia, 2 of 15 [13.3%]; without dementia, 6 of 26 [23.1%]; difference, −9.8 percentage points; women: with dementia, 6 of 35 [17.1%]; without dementia, 9 of 36 [25.0%]; difference, −7.9 percentage points) and sanitation (men: with dementia, 0 of 15; without dementia, 2 of 26 [7.7%]; difference, −7.7 percentage points; women: with dementia, 0 of 35; without dementia, 1 of 36 [2.8%]; difference, −2.8 percentage points). Similarly, a higher proportion of adults from all age groups were more deprived of education and health. Health deprivation was more likely after 60 years of age, particularly for adults with dementia. Conversely, unemployment was more likely among those aged 50 to 59 years, chiefly for individuals with dementia.

We estimated Spearman rank correlation coefficients between each pair of indicators of deprivation (eTable 3 in the Supplement). We found no evidence of a strong correlation between indicators, illustrating the absence of association between them except for type of housing, access to clean water, and sanitation. We kept those indicators as part of the living standards dimension of poverty but gave them each one-third the weight of other indicators in the equal nested weights measure to account for the risk of overrepresenting this specific dimension.^20^ This limited amount of overlap between indicators substantiates a multidimensional approach to poverty that does not rely on a unique welfare indicator of poverty, such as income, to represent all facets of poverty.

### Multidimensional Poverty

The prevalence of poverty varied across each group of adults based on the cutoff of the number of dimensions considered. When an individual was considered to be multidimensionally poor if deprived on only 1 dimension,^68^ 96.0% of older adults (48 of 50) with dementia as determined by both RUDAS and AD8, 87.0% of those (100 of 115) with dementia as determined by either RUDAS or AD8, and 82.3% of those (51 of 62) without dementia were deprived. Conversely, if we chose an intersectional approach in which being multidimensionally poor required being deprived in all 11 dimensions, no individual was multidimensionally poor; in fact, less than 1% of the sample (2 of 227 [0.9%]) were deprived in 7 dimensions. If multidimensional poverty was defined as being deprived in 4 dimensions, the proportions of poor adults with dementia and those without dementia were, respectively, 20% (33 of 165) and 9.7% (6 of 62) (eTable 4 in the Supplement).

**Figure 3.**
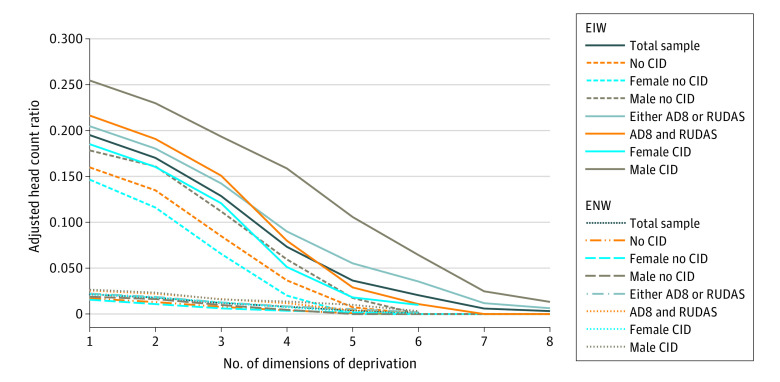
Adjusted Poverty Head Count for Both Weighting Structures (by Dementia Status and by Gender) For the 8-item Interview to Differentiate Aging and Dementia (AD8), cognitive impairment is considered present if a score of 2 or more is observed in the AD8 questions. For the Rowland Universal Dementia Assessment Scale (RUDAS), a score of 22 or less should be considered as possible cognitive impairment. CID indicates cognitive impairment disorder; EIW, equal indicator weight; and ENW, equal nested weight.

Adults who screened above the dementia threshold for both AD8 and RUDAS had a significantly higher adjusted head count ratio (Figure 3) for dimensions ranging between 1 and 3 (35.4% [(0.216-0.160)/0.160] for those deprived in 1 dimension, 41.5% [(0.191-0.135)/0.135] for those deprived in 2 dimensions, and 77.1% [(0.151-0.085)/0.085] for those deprived in 3 dimensions). This was also true for adults who screened above the dementia threshold for either AD8 or RUDAS for dimensions ranging between 1 and 5 (28.1% [(0.205-0.160)/0.160] for those deprived in 1 dimension, 33.6% [(0.180-0.135)/0.135] for those deprived in 2 dimensions, 67.3% [(0.142-0.085)/0.085] for those deprived in 3 dimensions, 145.8% [(0.090-0.037)/0.037] for those deprived in 4 dimensions, and 654.8% [0.055-0.007)/0.007] for those deprived in 5 dimensions). The mean deprivation share was higher among older adults with dementia for dimensions ranging between 1 and 5. This difference in the mean deprivation share is at the highest for deprivation in 2 dimensions (22% and 14% difference, respectively, in groups 1 and 2) (eTable 4 in the Supplement). The difference in the Multidimensional Poverty Index for deprivation in 4 dimensions between adults with and adults without dementia was 145.8% when screening for dementia with the AD8 and the RUDAS and 118.2% when screening for dementia with the AD8 or the RUDAS.

The adjusted head count ratio was higher for dimensions ranging between 1 and 6 for women with dementia compared with women without dementia and for dimensions ranging between 1 and 8 for men with dementia compared with men without dementia (Figure 3). No woman without dementia was poor with deprivation in more than 4 dimensions, and no man without dementia was poor with deprivation in more than 5 dimensions, whereas 3.6% of women with dementia (4 of 110) were poor with deprivation in 5 dimensions and 10.9% of men with dementia (6 of 55) were poor with deprivation in 6 dimensions. Overall, men with dementia were poorer, with deprivation in more dimensions, than women with dementia. The difference in the adjusted head count ratio between men with dementia and men without dementia is significant and reaches 166% for those deprived in 4 dimensions and 505% for those deprived in 5 dimensions.

Deprivations of education, health, and employment separately contributed, respectively, to more than 18%, 19%, and 10% (with 1 exception [8.4%] for those deprived in 3 dimensions) of the adjusted head count ratio for older adults with dementia compared with 16%, 13%, and 8% (with 1 exception [4%] for those deprived in 4 dimensions) for those without dementia for dimensions ranging between 1 and 4 (eTable 5 in the Supplement). Social cohesion contributed highly (>15%) to the adjusted head count ratio for older adults without dementia for dimensions ranging between 1 and 3.

The same 3 dimensions—deprivation of education, health, and employment—were the main contributors to the adjusted poverty head count poverty ratio for older men and women with or without dementia, for dimensions ranging between 1 and 4. Education contributed the most for both men and women with or without dementia, with the highest contribution for women with dementia (26.8% for those deprived in 1 dimension, 23.2% for those deprived in 2 dimensions, and 20.6% for those deprived in 3 dimensions). Employment contributed more to the adjusted head count ratio for both men and women with dementia compared with those without dementia. Poor health contributed more to the adjusted head count ratio for men without dementia, while deprivation of social participation contributed more to the adjusted head count ratio for women without dementia.

### Multivariable Regression Analysis

The odds of dementia were 2.31 (95% CI, 1.08-4.95) times higher for adults who were multidimensionally poor (those deprived in 4 dimensions) compared with those who were not multidimensionally poor, even after controlling for gender, marital status, age, and size of household (Table 2). Being a woman increased the relative odds of dementia by 2.03 (95% CI, 1.00-4.12), while living in a larger household increased the odds by 1.27 (95% CI, 1.05-1.53) for each additional household member. Age and marital status had no significant association with dementia. We tested the interaction between poverty and gender, between poverty and marital status, and between poverty and household size, and the results were not statistically significant.

**Table 2.** Logistic Regression Results of Association of Poverty, Gender, Age, Civil Status, and Household Size With Dementia

Characteristic	Unadjusted analysis		Adjusted analysis	
	OR (95% CI)	*P* value	OR (95% CI)	*P* value
No CID (base outcome)	NA	NA	NA	NA
CID defined by either RUDAS, AD8, or both				
Multidimensionally poor (reference: not poor)	1.96 (1.00-3.84)	.05	2.31 (1.08-4.95)	.03
Female (reference: male)	1.44 (0.79-2.63)	.23	2.03 (1.00-4.12)	.05
Living with partner (reference: living alone)	1.46 (0.79-2.70)	.22	1.76 (0.85-3.64)	.13
Age (continuous)	1.02 (0.98-1.05)	.43	1.01 (0.97-1.05)	.54
Household size (continuous)	1.29 (1.09-1.53)	.003	1.27 (1.05-1.53)	.001
Constant	NA	NA	0.13 (0.01-2.23)	.16

Abbreviations: AD8, 8-item Interview to Differentiate Aging and Dementia; CID, cognitive impairment disorder; NA, not applicable; OR, odds ratio; RUDAS, Rowland Universal Dementia Assessment Scale.

### Sensitivity Analysis

The multidimensional poverty calculation was repeated using the equal nested weight structure between the 7 dimensions, and the results remained unchanged (eTable 6 in the Supplement), including when stratified by gender (eTable 7 in the Supplement). Multidimensional poverty was found to be significantly higher for older adults with dementia compared with older adults without dementia for either AD8 or RUDAS screening for dimensions 1, 2, and 5; and for both AD8 and RUDAS screening for dimensions 1 through 4. The difference existed for other cutoffs but was not statistically significant. Women and men with dementia were poorer than women and men without dementia for any cutoff between 1 and 6 dimensions. Contributions of dimensions to the adjusted head count ratio consistently showed the prominence of education, health, and employment for older adults with or without dementia and for both genders (eTable 8 in the Supplement).

## Discussion

This study found that adults with dementia have a higher level of multidimensional poverty in an LMIC context. Considering 7 domains of social and environmental determinants of health (education, health status, employment, living standards, social participation, stigma, and psychological well-being), we found that exposure to multidimensional poverty was associated with dementia. Men with dementia were poorer and were deprived in a higher number of dimensions than women with dementia. However, being a man was protective against dementia, as previously reported.^69^ Living in a large household was also associated with a higher prevalence of dementia. This finding may be due to the fact that an older adult with declining independence or functioning may move back with his or her family, this older adult may receive less support, or fewer resources may be available to this older adult.^31^ Despite the fact that dementia prevalence increases with age, we did not find that age significantly strengthened the association with poverty. In addition, deprivation of education, health, and employment were identified as major contributors to multidimensional poverty, which constitutes an important indicator that social and environmental determinants of health are associated with dementia. Access to affordable and quality universal health care has been promoted as essential to reducing the effect of dementia.^70^ Moreover, policies promoting a fair economic arrangement and equal access in key sectors of society (eg, education and health care) may prevent health disparities over time.^71^ Alleviating multiple poverty indicators has the potential to slow the deterioration of cognitive functioning, which may advance interventions and prevention strategies in LMICs where the highest increase in dementia prevalence is expected but where limited research is taking place.

### Limitations

This study has some limitations. First, it was not possible to establish the direction of the association between poverty and dementia—poverty can be a cause as well as a consequence of dementia. Second, the size of the sample is limited, reducing the statistical power to further investigate the difference in multidimensional poverty by other sociodemographic characteristics of adults, such as age group or marital status. Third, because we could not obtain conventional biomarkers (eg, imaging and cerebrospinal fluid) or a formal assessment of cognitive functioning, we screened participants using the AD8 and the RUDAS. This approach did not establish a formal diagnosis of dementia; thus, we cannot conclusively exclude that the score obtained for both measures may be associated with the level of education or the socioeconomic status of the study participant. Fourth, 22.3% of participants in the sample were missing interviews, which might have introduced bias in our results because we cannot assume they were missing completely at random.

## Conclusions

Our study provides evidence for physicians, allied health professionals, and policy makers to consider daily stressors associated with multidimensional poverty and aging. This study offers some valuable insight into LMICs and what public policies (access to quality education, a strong workforce, and quality and free universal health care) could be prioritized that may be associated with dementia prevention and may reduce its effect on families and communities.^70^

## Notes

Supplement. **eTable 1.** Dimensions of Poverty and Indicators of Deprivation **eTable 2A.** Proportion of Participants Deprived in Each Dimension by Status of Dementia **eTable 2B.** Proportion of Participants Deprived in Each Dimension by Status of Dementia and Gender **eTable 2C.** Proportion of Participants Deprived in Each Dimension by Status of Dementia and Age **eTable 3.** Spearman Rank Correlations Between Dimensions of Deprivation **eTable 4.** Multidimensional Poverty Measures for Persons With and Without Dementia With Equal Indicators Weight **eTable 5.** Percentage Contribution of Each Dimension to Multidimensional Poverty by Dementia Status and by Gender With Equal Indicators Weight **eTable 6.** Multidimensional Poverty Measures for Persons With and Without Dementia With Equal Nested Weight **eTable 7.** Multidimensional Poverty Measures for Persons With and Without Dementia by Gender With Equal Nested Weight **eTable 8.** Percentage Contribution of Each Dimension to Multidimensional Poverty by Dementia Status and by Gender With Equal Nested Weight
