# Supplementary material for: Association of Multidimensional Poverty With Dementia in Adults Aged 50 Years or Older in South Africa
Source: JAMA Netw Open. Author manuscript; Available in PMC 2022 May 29. (PMC8956981; doi:10.1001/jamanetworkopen.2022.4160)
Supplement: South Africa Tables — eTable 1. Dimensions of Poverty and Indicators of Deprivation eTable 2A. Proportion of Participants Deprived in Each Dimension by Status of Dementia eTable 2B. Proportion of Participants Deprived in Each Dimension by Status of Dementia and Gender eTable 2C. Proportion of Participants Deprived in Each Dimension by Status of Dementia and Age eTable 3. Spearman Rank Correlations Between Dimensions of Deprivation eTable 4. Multidimensional Poverty Measures for Persons With and Without Dementia With Equal Indicators Weight eTable 5. Percentage Contribution of Each Dimension to Multidimensional Poverty by Dementia Status and by Gender With Equal Indicators Weight eTable 6. Multidimensional Poverty Measures for Persons With and Without Dementia With Equal Nested Weight eTable 7. Multidimensional Poverty Measures for Persons With and Without Dementia by Gender With Equal Nested Weight eTable 8. Percentage Contribution of Each Dimension to Multidimensional Poverty by Dementia Status and by Gender With Equal Nested Weight [file NIHMS1796677-supplement-South_Africa_Tables.pdf]

Supplementary Online Content

Trani JF, Moodley J, Maw MTT, Babulal GM. Association of multidimensional poverty with dementia in adults aged 50 years or older in South Africa. *JAMA Netw Open.* 2022;5(3):e224160. doi:10.1001/jamanetworkopen.2022.4160

eTable 1. Dimensions of Poverty and Indicators of Deprivation
eTable 2A. Proportion of Participants Deprived in Each Dimension by Status of Dementia
eTable 2B. Proportion of Participants Deprived in Each Dimension by Status of Dementia and Gender
eTable 2C. Proportion of Participants Deprived in Each Dimension by Status of Dementia and Age
eTable 3. Spearman Rank Correlations Between Dimensions of Deprivation
eTable 4. Multidimensional Poverty Measures for Persons With and Without Dementia With Equal Indicators Weight
eTable 5. Percentage Contribution of Each Dimension to Multidimensional Poverty by Dementia Status and by Gender With Equal Indicators Weight
eTable 6. Multidimensional Poverty Measures for Persons With and Without Dementia With Equal Nested Weight
eTable 7. Multidimensional Poverty Measures for Persons With and Without Dementia by Gender With Equal Nested Weight
eTable 8. Percentage Contribution of Each Dimension to Multidimensional Poverty by Dementia Status and by Gender With Equal Nested Weight

This supplementary material has been provided by the authors to give readers additional information about their work.

© 2022 Trani JF et al. *JAMA Network Open.*

eTable 1. Dimensions of Poverty and Indicators of Deprivation

Dimensions	Indicators	Deprivation cutoff (Deprived if…)
**Education**		
1. Adult educational attainment	What is HH highest Education Level?	Less than 8 grade and no schooling
**Health Status**		
2. Activities limitation and functioning problem	Any Difficulties you may have doing certain activities because of a health problem?	Participant answered " a lot of difficulty/ cannot do at all" in any of 6 Questions of Washington Group for disability statistics: Seeing, Hearing, walking, concentrating, dressing, communicating
**Household-level Material well-being/ Living Standards**		
3. Dwelling type/ Housing	In what type of dwelling do you currently live?	Housing type is not "House or brick structure on a separate stand or yard"
4. Water source	What is the main source of drinking water for your current household?	The household does not have "Piped drinking water in dwelling"
5. Energy source	What is the main source of energy that this household uses for cooking, heating and lighting?	The household does not use “Electricity"
6. Sanitation	What type of toilet does your current household use?	The household does not "Own flush toilet inside the house" or "Own flush toilet outside the house"
**Employment Status**		
7. Employment	Which one of the following best describes what you are currently doing?, Are you currently looking for job and reason not looking for job	Deprived on Employment if participant said "yes to currently looking for a job" or "Participant was not looking for a job because he/she has the following reason: I became discouraged, I could not afford the cost of looking for work, The wages were too low"
7.B. Employment (Adapted Version)	Which one of the following best describes what you are currently doing?, Are you currently looking for job and reason not looking for job	Deprived on Employment is decided on case by case depends on the individual situation.<br>Example, if Participant answered not looking for a job because he/she is too old to work, we look at the participant's age, health status condition and current employment condition. Older adults with disability may have willing to work but there is no job opportunity for them

© 2022 Trani JF et al. *JAMA Network Open.*

**Social Participation**		
8. Social Cohesion	Do you belong to any of the following social groups?	Not belong to any social groups
**Fair treatment (Safety?)**		
9. Stigma (Experienced unfair treatment)	Use DISC Questionnaires	Deprived if stigma score is >1.5 (had at least moderate discrimination)
**Psychological well being**		
10. Mental Well Being (Depression)	Use 10 CES-D questionnaires	Deprived if CES-D score >=16
11. Self esteem	Rosenberg's Self-Esteem Scale	The scale ranges from 0-30. Scores between 15 and 25 are within normal range; scores below 15 suggest low self-esteem.

Notes: HH: Household; DISC: Discrimination and Stigma Scale; CES-D: Center for Epidemiologic Studies - Depression Scale.

© 2022 Trani JF et al. *JAMA Network Open.*

eTable 2A. Proportion of Participants Deprived in Each Dimension by Status of Dementia

	**Participants deprived in each dimension, No. (%)**				
**Dimensions**	**No CID[a]**	**Either AD8 or RUDAS[b]**	**Both AD8 or RUDAS[c]**	**All**	***P* value**
Education	25 (40.32)	64 (55.65)	31 (62)	120 (52.86)	0.051
Health status	15 (24.19)	31 (26.96)	23 (46)	69 (30.4)	0.022
Employment	9 (14.52)	39 (33.91)	14 (28)	62 (27.31)	0.023
Housing	4 (6.45)	12 (10.43)	5 (10)	21 (9.25)	0.669
Water source	15 (24.19)	23 (20)	8 (16)	46 (20.26)	0.56
Energy source	1 (1.61)	6 (5.22)	1 (2)	8 (3.52)	0.372
Sanitation	3 (4.84)	9 (7.83)	0 (0)	12 (5.29)	0.117
Social cohesion	18 (29.03)	24 (20.87)	16 (32)	58 (25.55)	0.245
Unfair treatment	2 (3.23)	4 (3.48)	2 (4)	8 (3.52)	0.975
Depression	9 (14.52)	25 (21.74)	7 (14)	41 (18.06)	0.344
Self-esteem	8 (12.9)	22 (19.13)	12 (24)	42 (18.5)	0.313

Abbreviations: CID, Cognitive Impairment Disorder, AD8, AD8 Dementia Screening Interview Questionnaires: *The Washington University Dementia Screening Test, Cognitive Impairment present if ADQ >=2 in total of AD 8 questions*; RUDAS, *The Rowland Universal Dementia Assessment Scale, Deprived of Any score of 22 or less should be considered as possible cognitive impairment (Total 30 RUDAS) Questions 2004*

[a] No Cognitive impairment defined by both AD8 and RUDAS

[b] Cognitive impairment defined by either of AD8 scores or RUDAS scores

[c] Cognitive impairment defined by both AD8 scores and RUDAS scores

© 2022 Trani JF et al. *JAMA Network Open.*

**eTable 2B.** Proportion of Participants Deprived in Each Dimension by Status of Dementia and Gender								
	**Male No. (%)**				**Female No. (%)**			
**Dimensions**	**No CID[a]**	**Either AD8 or RUDAS[b]**	**Both AD8 or RUDAS[c]**	***P* value**	**No CID[a]**	**Either AD8 or RUDAS[b]**	**Both AD8 or RUDAS[c]**	***P* value**
Education	12 (46.15)	25 (62.5)	10 (66.67)	0.318	13 (36.11)	39 (52)	21 (60)	0.117
Health status	9 (34.62)	11 (27.5)	8 (53.33)	0.2	6 (16.67)	20 (26.67)	15 (42.86)	0.045
Employment	5 (19.23)	17 (42.5)	6 (40)	0.135	4 (11.11)	22 (29.33)	8 (22.86)	0.104
Housing	3 (11.54)	9 (22.5)	2 (13.33)	0.466	1 (2.78)	3 (4)	3 (8.57)	0.468
Water source	6 (23.08)	12 (30)	2 (13.33)	0.431	9 (25)	11 (14.67)	6 (17.14)	0.409
Energy source	1 (3.85)	4 (10)	0 (0)	0.326	0 (0)	2 (2.67)	1 (2.86)	0.604
Sanitation	2 (7.69)	7 (17.5)	0 (0)	0.147	1 (2.78)	2.67 (7)	0 (0)	0.617
Social cohesion	6 (23.08)	7 (17.5)	4 (26.67)	0.721	12 (33.33)	17 (22.67)	12 (34.29)	0.325
Unfair treatment	0 (0)	1 (2.5)	1 (6.67)	0.416	2 (5.56)	3 (4)	1 (2.86)	0.847
Depression	3 (11.54)	13 (32.5)	3 (20)	0.137	6 (16.67)	12 (16)	4 (11.43)	0.785
Self-esteem	4 (15.38)	8 (20)	4 (26.67)	0.682	4 (11.11)	14 (18.67)	8 (22.86)	0.417

Abbreviations: CID, Cognitive Impairment Disorder, AD8, AD8 Dementia Screening Interview Questionnaires: The Washington University Dementia Screening Test, Cognitive Impairment present if ADQ >=2 in total of AD 8 questions; RUDAS, The Rowland Universal Dementia Assessment Scale, Deprived of Any score of 22 or less should be considered as possible cognitive impairment (Total 30 RUDAS) Questions 2004

[a] No Cognitive impairment defined by both AD8 and RUDAS

[b] Cognitive impairment defined by either of AD8 scores or RUDAS scores

[c] Cognitive impairment defined by both AD8 scores and RUDAS scores

© 2022 Trani JF et al. *JAMA Network Open.*

eTable 2C. Proportion of Participants Deprived in Each Dimension by Status of Dementia and Age

	50 – 59 no (%)				60-69 no (%)				>70 no (%)			
**Dimensions**	**No CID[a]**	**Either AD8 or RUDAS[b]**	**Both AD8 and RUDAS[c]**	***P* value**	**No CID[a]**	**Either AD8 or RUDAS[b]**	**Both AD8 and RUDAS[c]**	***P* value**	**No CID[a]**	**Either AD8 or RUDAS[b]**	**Both AD8 and RUDAS[c]**	***P* value**
Education	5 (23.81)	19 (45.24)	9 (52.94)	0.144	13 (46.43)	28 (56)	13 (68.42)	0.355	7 (53.85)	17 (73.91)	9 (64.29)	0.469
Health status	4 (19.05)	8 (19.05)	6 (35.29)	0.363	5 (17.86)	13 (26)	10 (52.63)	0.029	6 (46.15)	10 (43.48)	7 (50)	0.928
Employment	7 (33.33)	22 (52.38)	8 (47.06)	0.359	1 (3.57)	13 (26)	4 (21.05)	0.048	1 (7.69)	4 (17.39)	2 (14.29)	0.722
Housing	3 (14.29)	6 (14.29)	4 (23.53)	0.657	1 (3.57)	5 (10)	1 (5.26)	0.537	0 (0)	1 (4.35)	0 (0)	0.549
Water source	8 (38.1)	12 (28.57)	5 (29.41)	0.732	3 (10.71)	6 (12)	2 (10.53)	0.978	4 (30.77)	5 (21.74)	1 (7.14)	0.296
Energy source	0 (0)	2 (4.76)	1 (5.88)	0.562	1 (3.57)	2 (4)	0 (0)	0.682	0 (0)	2 (8.7)	0 (0)	0.294
Sanitation	3 (14.29)	3 (7.14)	0 (0)	0.249	0 (0)	5 (10)	0 (0)	0.084	0 (0)	1 (4.35)	0 (0)	0.549
Social cohesion	0 (0)	0 (0)	0 (0)	NA	6 (21.43)	7 (14)	5 (26.32)	0.45	12 (92.31)	17 (73.91)	11 (78.57)	0.41
Unfair treatment	0 (0)	2 (4.76)	1 (5.88)	0.562	2 (7.14)	2 (4)	1 (5.26)	0.834	0 (0)	0 (0)	0 (0)	NA
Depression	5 (23.81)	8 (19.05)	3 (17.65)	0.872	3 (10.71)	11 (22)	3 (15.79)	0.442	1 (7.69)	6 (26.09)	1 (7.14)	0.199
Self-esteem	6 (28.57)	4 (9.52)	6 (35.29)	0.042	2 (7.14)	11 (22)	6 (31.58)	0.097	0 (0)	7 (30.43)	0 (0)	0.08

Abbreviations: CID, Cognitive Impairment Disorder, AD8, AD8 Dementia Screening Interview Questionnaires: The Washington University Dementia Screening Test, Cognitive Impairment present if ADQ >=2 in total of AD 8 questions; RUDAS, The Rowland Universal Dementia Assessment Scale, Deprived of Any score of 22 or less should be considered as possible cognitive impairment (Total 30 RUDAS) Questions 2004

[a] No Cognitive impairment defined by both AD8 and RUDAS

[b] Cognitive impairment defined by either of AD8 scores or RUDAS scores

[c] Cognitive impairment defined by both AD8 scores and RUDAS scores

© 2022 Trani JF et al. *JAMA Network Open.*

eTable 3. Spearman Rank Correlations Between Dimensions of Deprivation

	Education	Employment	Health	Housing	Water	Energy	Sanitation	Inclusion	Stigma	Depression	Self-esteem
Education	1										
Employment	-0.0352	1									
Health	0.0676	0.0248	1								
Housing	0.0578	0.2138*	-0.0457	1							
Water	0.0369	0.1091	0.0004	0.5199*	1						
Energy	0.0369	0.0973	-0.0224	0.2688*	0.2008*	1					
Sanitation	0.1048	0.0761	-0.0277	0.6040*	0.3217*	0.4887*	1				
Inclusion	0.1283	-0.2458*	0.3156*	-0.1522*	0.0062	-0.0024	-0.0933	1			
Stigma	0.0369	0.0437	0.1334*	-0.061	0.0225	0.093	0.0616	-0.0572	1		
Depression	-0.1072	-0.0051	0.2374*	0.0477	0.0482	-0.0276	0.0426	-0.0913	0.0966	1	
Self-esteem	0.0181	-0.1648*	0.2524*	0.0436	0.042	-0.0295	-0.0112	0.007	0.0935	0.3661*	1

Note: * indicate correlation significant at p<0.05

© 2022 Trani JF et al. *JAMA Network Open.*

eTable 4. Multidimensional Poverty Measures for Persons With and Without Dementia With Equal Indicators Weight

	All together			NO CID			Either AD8 or RUDAS above threshold					% difference	% difference	Both AD8 or RUDAS above threshold					% difference	% difference
K	H	A	M0	H	A	M0	H	A	M0	T value	P value	in A	in M0*	H	A	M0	T value	P value	in A	in M0*
1	0.877	0.222	0.195	0.823	0.194	0.160	0.870	0.235	0.205	-1.994	0.048	21.18	28.12	0.960	0.225	0.216	-2.509	0.014	16.0	35.38
2	0.604	0.282	0.170	0.548	0.246	0.135	0.600	0.300	0.180	-1.752	0.082	22.12	33.61	0.680	0.281	0.191	-2.054	0.042	14.13	41.52
3	0.374	0.343	0.129	0.274	0.310	0.085	0.391	0.364	0.142	-2.058	0.041	17.24	67.32	0.460	0.328	0.151	-2.211	0.029	5.77	77.45
4	0.172	0.427	0.073	0.097	0.379	0.037	0.200	0.451	0.090	-2.057	0.041	18.96	145.84	0.200	0.400	0.080	-1.650	0.102	5.60	118.24
5	0.070	0.517	0.036	0.016	0.455	0.007	0.104	0.530	0.055	-2.217	0.028	16.67	654.78	0.060	0.485	0.029	-1.286	0.201	6.67	296.80
6	0.035	0.580	0.020	0	.	0	0.061	0.584	0.036	-1.980				0.020	0.545	0.011	-1.115	0.267		
7	0.009	0.682	0.006	0	.	0	0.017	0.682	0.012	-1.039				0	.	0				
8	0.004	0.727	0.003	0	.	0	0.009	0.727	0.006	-0.733				0	.	0				

© 2022 Trani JF et al. *JAMA Network Open.*

eTable 5. Percentage Contribution of Each Dimension to Multidimensional Poverty by Dementia Status and by Gender With Equal Indicators Weight

	k	Educa-tion	Health status	Employ-ment	Housing	Water source	Energy source	Sanitation	Social cohesion	Unfair treatment	Depression	Self-esteem
**No dementia**	1	30.86	11.11	18.52	1.23	4.01	0.00	0.93	22.22	2.47	4.94	3.70
	2	28.80	11.20	24.00	1.20	3.20	0.00	0.40	22.40	1.60	4.00	3.20
	3	21.62	10.81	29.73	0.68	4.05	0.00	0.68	24.32	2.70	4.05	1.35
	4	25.97	5.19	31.17	0.00	1.30	0.00	0.00	31.17	5.19	0.00	0.00
**Either AD8 or RUDAS**	1	32.49	19.80	15.74	1.52	2.66	0.76	1.14	12.18	2.03	6.09	5.58
	2	27.75	18.70	17.50	1.81	3.02	0.90	1.36	13.88	2.41	6.64	6.03
	3	26.01	15.25	20.63	1.57	3.59	1.35	1.57	15.25	3.59	4.93	6.28
	4	25.33	16.00	22.67	1.33	2.67	1.67	1.67	14.67	4.00	5.33	4.67
	5	25.26	14.74	18.95	1.05	2.11	1.58	1.58	16.84	4.21	7.37	6.32
	6	22.54	16.90	22.54	1.41	2.82	1.41	1.41	16.90	0.00	8.45	5.63
**Both AD8 & RUDAS**	1	31.47	14.21	23.35	1.27	1.78	0.00	0.00	16.24	2.03	3.55	6.09
	2	29.33	12.90	26.98	1.47	1.76	0.00	0.00	14.08	2.35	4.11	7.04
	3	28.69	9.56	27.09	1.59	1.99	0.00	0.00	15.94	3.19	4.78	7.17
	4	28.73	6.63	30.94	0.55	1.10	0.00	0.00	17.68	4.42	4.42	5.52
	5	26.23	13.11	26.23	1.64	3.28	0.00	0.00	6.56	13.11	3.28	6.56
	6	23.53	0.00	23.53	0.00	5.88	0.00	0.00	0.00	23.53	11.76	11.76
**Male without dementia**	1	31.79	13.25	23.84	1.99	3.97	0.00	1.32	15.89	0.00	3.97	3.97
	2	30.53	12.21	27.48	1.53	3.05	0.00	0.76	15.27	0.00	4.58	4.58
	3	20.25	15.19	35.44	1.27	3.80	0.00	1.27	15.19	0.00	5.06	2.53
**Male with dementia**	1	31.11	20.44	16.89	2.44	3.11	0.89	1.56	9.78	1.78	6.67	5.33
	2	27.41	19.29	18.27	2.79	3.55	1.02	1.78	10.15	2.03	7.61	6.09
	3	26.77	16.36	19.33	2.60	4.09	1.49	1.86	10.41	2.97	6.69	7.43
	4	25.97	13.85	22.51	2.16	3.03	1.73	2.16	12.12	3.46	6.06	6.93
	5	25.73	14.04	21.05	1.75	2.92	1.17	1.75	14.04	4.68	5.85	7.02

© 2022 Trani JF et al. *JAMA Network Open.*

	6	22.22	14.81	22.22	1.85	3.70	0.00	1.85	14.81	0.00	11.11	7.41
**Female without dementia**	1	30.06	9.25	13.87	0.58	4.05	0.00	0.58	27.75	4.62	5.78	3.47
	2	26.89	10.08	20.17	0.84	3.36	0.00	0.00	30.25	3.36	3.36	1.68
	3	23.19	5.80	23.19	0.00	4.35	0.00	0.00	34.78	5.80	2.90	0.00
**Female with dementia**	1	32.79	16.39	19.13	0.82	1.91	0.27	0.27	15.85	2.19	4.37	6.01
	2	28.85	15.08	22.30	0.98	1.97	0.33	0.33	16.39	2.62	4.59	6.56
	3	27.10	11.21	25.23	0.93	2.34	0.47	0.47	18.69	3.74	3.74	6.07
	4	27.20	11.20	28.80	0.00	1.20	0.40	0.00	19.20	4.80	4.00	3.20
	5	25.00	15.00	20.00	0.00	1.25	1.25	0.00	15.00	10.00	7.50	5.00
	6	23.53	11.76	23.53	0.00	2.94	2.94	0.00	11.76	11.76	5.88	5.88

Notes: AD8: AD8 Dementia Screening Interview Questionnaires: The Washington University Dementia Screening Test, Cognitive Impairment present if ADQ >=2 in total of AD 8 questions; RUDAS, The Rowland Universal Dementia Assessment Scale, Deprived of Any score of 22 or less should be considered as possible cognitive impairment (Total 30 RUDAS)

© 2022 Trani JF et al. *JAMA Network Open.*

eTable 6. Multidimensional Poverty Measures for Persons With and Without Dementia With Equal Nested Weight

	**All together**			**NO CID**			**Either AD8 or RUDAS above threshold**			**T value**	**P value**	**% difference**	**Both AD8 or RUDAS above threshold**					**% difference**
K	H	A	M0	H	A	M0	H	A	M0			in M0*	H	A	M0	T value	P value	in M0*
1	0.828	0.026	0.022	0.726	0.023	0.017	0.843	0.026	0.022	-2.272	0.024	31.12	0.920	0.028	0.026	-3.296	0.001	50.79
2	0.555	0.032	0.018	0.435	0.030	0.013	0.574	0.033	0.019	-2.045	0.042	42.98	0.660	0.034	0.022	-2.851	0.005	69.14
3	0.308	0.039	0.012	0.210	0.037	0.008	0.313	0.040	0.013	-1.695	0.092	62.47	0.420	0.039	0.016	-2.572	0.012	110.30
4	0.181	0.044	0.008	0.097	0.042	0.004	0.183	0.046	0.008	-1.709	0.089	110.05	0.280	0.042	0.012	-2.560	0.012	191.48
5	0.075	0.051	0.004	0.016	0.052	0.001	0.104	0.051	0.005	-2.145	0.033	540.22	0.080	0.050	0.004	-1.590	0.115	372.75
6	0.022	0.057	0.001	0.000	.	0.000	0.035	0.058	0.002	-1.485	0.140		0.020	0.055	0.001	-1.115	0.267	

© 2022 Trani JF et al. *JAMA Network Open.*

eTable 7. Multidimensional Poverty Measures for Persons With and Without Dementia by Gender With Equal Nested Weight

	**Female no CID**			**Female CID**					**% difference**	**Male no CID**			**Male CID**					**% difference**
K	H	A	M0	H	A	M0	T value	P value	in M0*	H	A	M0	H	A	M0	T value	P value	in M0*
1	0.722	0.022	0.016	0.836	0.026	0.022	-2.232	0.027	38.48	0.731	0.026	0.019	0.927	0.029	0.027	-2.123	0.037	40.88
2	0.361	0.030	0.011	0.564	0.032	0.018	-2.247	0.026	67.76	0.538	0.030	0.016	0.673	0.035	0.023	-1.583	0.118	42.18
3	0.167	0.037	0.006	0.336	0.038	0.013	-1.916	0.057	103.00	0.269	0.037	0.010	0.364	0.044	0.016	-1.239	0.219	60.97
4	0.083	0.043	0.004	0.173	0.043	0.007	-1.267	0.207	104.55	0.115	0.040	0.005	0.291	0.047	0.014	-1.944	0.055	195.14
5	0.028	0.052	0.001	0.045	0.052	0.002	-0.460	0.646	63.64	0		0	0.200	0.050	0.010	-2.499	0.015	
6	0		0	0.018	0.055	0.001	-0.811	0.419		0		0	0.055	0.058	0.003	-1.208	0.231	

© 2022 Trani JF et al. *JAMA Network Open.*

eTable 8. Percentage Contribution of Each Dimension to Multidimensional Poverty by Dementia Status and by Gender With Equal Nested Weight

	k	Education	Health status	Employ-ment	Housing	Water source	Energy source	Sanitation	Social cohesion	Unfair treatment	Depression	Self-esteem
No dementia	1	30.86	11.11	18.52	1.23	4.01	0.00	0.93	22.22	2.47	4.94	3.70
	2	28.80	11.20	24.00	1.20	3.20	0.00	0.40	22.40	1.60	4.00	3.20
	3	21.62	10.81	29.73	0.68	4.05	0.00	0.68	24.32	2.70	4.05	1.35
	4	25.97	5.19	31.17	0.00	1.30	0.00	0.00	31.17	5.19	0.00	0.00
Either AD8 or RUDAS	1	32.49	19.80	15.74	1.52	2.66	0.76	1.14	12.18	2.03	6.09	5.58
	2	27.75	18.70	17.50	1.81	3.02	0.90	1.36	13.88	2.41	6.64	6.03
	3	26.01	15.25	20.63	1.57	3.59	1.35	1.57	15.25	3.59	4.93	6.28
	4	25.33	16.00	22.67	1.33	2.67	1.67	1.67	14.67	4.00	5.33	4.67
	5	25.26	14.74	18.95	1.05	2.11	1.58	1.58	16.84	4.21	7.37	6.32
	6	22.54	16.90	22.54	1.41	2.82	1.41	1.41	16.90	0.00	8.45	5.63
Both AD8 & RUDAS	1	31.47	14.21	23.35	1.27	1.78	0.00	0.00	16.24	2.03	3.55	6.09
	2	29.33	12.90	26.98	1.47	1.76	0.00	0.00	14.08	2.35	4.11	7.04
	3	28.69	9.56	27.09	1.59	1.99	0.00	0.00	15.94	3.19	4.78	7.17
	4	28.73	6.63	30.94	0.55	1.10	0.00	0.00	17.68	4.42	4.42	5.52
	5	26.23	13.11	26.23	1.64	3.28	0.00	0.00	6.56	13.11	3.28	6.56
	6	23.53	0.00	23.53	0.00	5.88	0.00	0.00	0.00	23.53	11.76	11.76
Male without dementia	1	31.79	13.25	23.84	1.99	3.97	0.00	1.32	15.89	0.00	3.97	3.97
	2	30.53	12.21	27.48	1.53	3.05	0.00	0.76	15.27	0.00	4.58	4.58
	3	20.25	15.19	35.44	1.27	3.80	0.00	1.27	15.19	0.00	5.06	2.53
Male with dementia	1	31.11	20.44	16.89	2.44	3.11	0.89	1.56	9.78	1.78	6.67	5.33
	2	27.41	19.29	18.27	2.79	3.55	1.02	1.78	10.15	2.03	7.61	6.09
	3	26.77	16.36	19.33	2.60	4.09	1.49	1.86	10.41	2.97	6.69	7.43

© 2022 Trani JF et al. *JAMA Network Open.*

	4	25.97	13.85	22.51	2.16	3.03	1.73	2.16	12.12	3.46	6.06	6.93
	5	25.73	14.04	21.05	1.75	2.92	1.17	1.75	14.04	4.68	5.85	7.02
	6	22.22	14.81	22.22	1.85	3.70	0.00	1.85	14.81	0.00	11.11	7.41
Female without dementia	1	30.06	9.25	13.87	0.58	4.05	0.00	0.58	27.75	4.62	5.78	3.47
	2	26.89	10.08	20.17	0.84	3.36	0.00	0.00	30.25	3.36	3.36	1.68
	3	23.19	5.80	23.19	0.00	4.35	0.00	0.00	34.78	5.80	2.90	0.00
Female with dementia	1	32.79	16.39	19.13	0.82	1.91	0.27	0.27	15.85	2.19	4.37	6.01
	2	28.85	15.08	22.30	0.98	1.97	0.33	0.33	16.39	2.62	4.59	6.56
	3	27.10	11.21	25.23	0.93	2.34	0.47	0.47	18.69	3.74	3.74	6.07
	4	27.20	11.20	28.80	0.00	1.20	0.40	0.00	19.20	4.80	4.00	3.20
	5	25.00	15.00	20.00	0.00	1.25	1.25	0.00	15.00	10.00	7.50	5.00
	6	23.53	11.76	23.53	0.00	2.94	2.94	0.00	11.76	11.76	5.88	5.88

© 2022 Trani JF et al. *JAMA Network Open.*
